# Targeting tumour-reprogrammed myeloid cells: the new battleground in cancer immunotherapy

**DOI:** 10.1007/s00281-022-00965-1

**Published:** 2022-09-26

**Authors:** Francesco De Sanctis, Annalisa Adamo, Stefania Canè, Stefano Ugel

**Affiliations:** Immunology Section, University Hospital and Department of Medicine, University of Verona, 37134 Verona, Italy

**Keywords:** Myeloid-derived suppressor cells (MDSC), Cancer, Tumour microenvironment (TME), Inflammation, Cancer immunotherapy

## Abstract

Tumour microenvironment is a complex ecosystem in which myeloid cells are the most abundant immune elements. This cell compartment is composed by different cell types, including neutrophils, macrophages, dendritic cells, and monocytes but also unexpected cell populations with immunosuppressive and pro-tumour roles. Indeed, the release of tumour-derived factors influences physiological haematopoiesis producing unconventional cells with immunosuppressive and tolerogenic functions such as myeloid-derived suppressor cells. These pro-tumour myeloid cell populations not only support immune escape directly but also assist tumour invasion trough non-immunological activities. It is therefore not surprising that these cell subsets considerably impact in tumour progression and cancer therapy resistance, including immunotherapy, and are being investigated as potential targets for developing a new era of cancer therapy. In this review, we discuss emerging strategies able to modulate the functional activity of these tumour-supporting myeloid cells subverting their accumulation, recruitment, survival, and functions. These innovative approaches will help develop innovative, or improve existing, cancer treatments.

## Introduction

Advanced understanding of immune system regulation circuits and biological insights of tumour-immune system interplay completely revolutionised the concept of cancer therapy. An example of this is the paramount success of immune checkpoint therapy (ICT) based on antibody-dependent targeting of T cell functional modulators like cytotoxic T lymphocyte antigen 4 (CTLA-4) and programmed cell death protein 1 (PD-1) that relies on cancer restriction through the activation of the host immune system and resulted in significant improved clinical benefits in many type of solid tumours [1–3]. However, ICT does not work yet as single agent in patients affected by tumours with specific histology and genetic features (e.g. in pancreatic cancer and glioblastoma) [4]. Moreover, even in tumour characterised by high mutation burden, such as melanoma and breast cancer, ICT improved the clinical outcome only in a small fraction of treated patients [1]. Numerous factors regulate the dynamic process of tumour immunity and response to immune checkpoint blockade that can be broadly categorised in two main classes: tumour-intrinsic and tumour-extrinsic factors [2, 5].

Over the tumour evolution, cancer cells acquire several mutations leading the expression of proteins with an altered folding or mutated encoding gene sequences that allow to generate new immunogenic peptides able to activate a specific immune reaction. These neo-antigens are able to evoke a potential tumour-restricted response since they are distinct from self-antigens [6]. Conversely, genetic and epigenetic modifications leading alterations of both antigen presentation machinery [7] and signal transduction pathways, such as interferon (IFN)γ-signalling defects [8], negatively influence the responsiveness of ICT. Tumour-extrinsic factors of immune resistance are dependent on tumour-microenvironment (TME) components. Indeed tumour can be considered a complex tissue in which neoplastic cells, immune cells, vascular components (e.g. endothelial cells), fibroblasts, and matrix interplay defines the fate of ICT. For instance, the CD8^+^ T cell localisation at tumour margins and within the tumour prior to ICT correlated positively with a robust response to immunotherapy [9], whilst regulatory T lymphocytes (Tregs) blunting the effector immune functions contributed to the clinical failure of ICT [10]. However, the most pervasive mechanism activated by tumours to alter the immune response in TME is the induction of an emergency haematopoiesis pushing the accumulation of myeloid cells with immunosuppressive functions and pro-inflammatory properties, such as myeloid-derived suppressor cells (MDSCs) [11, 12]. Indeed, these tumour-reprogrammed myeloid cells have the ability to support tumour progression by assisting tumour cell survival, angiogenesis, and metastatic process [13–15]. Collectively, tumour cells hijack both innate and adaptive immune resistance mechanisms to avoid immune-based clearance [16]. Accordingly, immune suppression, inflammation, abnormal differentiation, and function of myeloid cells are enlisted as hallmarks of cancer [17].

Here we review evidences indicating that targeting tumour-reprogrammed myeloid cells may be beneficial in promoting response to ICT. To develop more effective myeloid cell-targeted therapies is indeed mandatory to combine data of single-cell transcriptome, metabolic, and epigenetic profiles to pinpoint complex relationships between myeloid cells and other TME components. We think that immunotherapy should be considered an immune system rather than a cancer treatment and, therefore, any improvement will depend on overcoming the gaps in understanding the biology of TME immune components with particular focus on myeloid cells.

## Myeloid cells in tumour microenvironment

Immune cell heterogeneity entangles TME immune profiling [18]. In this context, myeloid cells represent the most abundant and functionally plastic immune components promoting both tumour recognition and escape. Indeed, myeloid cells rapidly infiltrate early neoplastic lesions and may dictate the fate of tumours by supporting either T cell-mediated killing by acting as professional tumour antigen-presenting cells or promoting immune arrest and cancer progression by inhibiting both adaptive and innate immunity. Tumour-associated macrophages (TAMs), MDSCs, tumour-associated neutrophils (TANs), and dendritic cells (DCs) are major tumour-infiltrating myeloid cells (TIMs) [19]. Myeloid cells identified in tumours have different ontogeny (i.e. bone marrow [BM]-derived or tissue resident cells) and are characterised by a plastic phenotype that can be shaped by cytokines and other soluble factors. Tumours support the emergency myelopoiesis favouring the generation of unconventional mature and immature myeloid cells endowed with tumour-promoting activities [20, 21]. For instance, a significant portion of tumour-infiltrating myeloid cells are of erythroid origin [22], highlighting how tumour reprograms myeloid cell differentiation. In this scenario, TAMs and MDSCs represent the ultimate commitment of the tumour-dependent myeloid-cell reprogramming [23, 24] and will be the focus of this review. For more detailed information on other tumour-infiltrating myeloid cells, please refer to the following manuscripts [25–29].

TAMs can be polarised towards an inflammatory (M1-like) or anti-inflammatory (M2-like) phenotype which supports immune control or immune evasion of neoplastic cells, respectively [30]. Notably, M1/M2 macrophage dichotomy is an oversimplification of the heterogeneity of macrophages that occurs in vivo [31]. TAMs can be polarised towards an inflammatory (M1-like) or anti-inflammatory (M2-like) phenotype which supports immune control or immune evasion of neoplastic cells, respectively. Notably, M1/M2 macrophage dichotomy is an oversimplification of the heterogeneity of macrophages that occurs in vivo. Indeed, M1- and M2-polarised macrophages should be viewed as the extremes of macrophage plasticity since TAM with identifiable M1 or M2 polarisation do not really exist in the tumours, instead being represented by TAM with mixed characteristics. However, it is interesting that this M1/M2 macrophage classification may explain the correlation between TME-infiltrating TAMs and patient outcome [32]. M1-TAMs are characterised by sustained phagocytosis and enhanced anti-tumour inflammatory reactions. This cell subset supports T cell activation by expressing co-stimulatory molecules (i.e. CD80, CD86) and high levels of major histocompatibility complex class II (MHCII) molecules. Instead, alternatively activated M2-TAMs support tumour cell survival, angiogenesis, and invasion [21]. These macrophages are characterised by higher expression of CD163 and CD206 both in humans and mice [33]. M2-TAMs can promote tumour progression by both soluble mediators (e.g. Arginase [Arg]1-derived products and transforming growth factor beta [TGFβ] release) and surface receptors (e.g. expression of programmed death-ligand 1 [PD-L1]), resulting in suppression of anti-tumour response [21]. Moreover, these cells actively support metastatic process by remodelling the extracellular matrix. Indeed, we recently showed that disabled homolog 2 mitogen-responsive phosphoprotein (DAB2)-expressing M2-TAMs play central role in lung metastasis formation by remodelling tissue matrix components [34]. Clinically, patients with tumour enriched in macrophages, especially the ones with pro-angiogenic features, have a poor prognosis and reduced overall survival [26, 31, 35–37]. TAMs were also reported to mediate chemotherapy resistance in different cancer settings by activating anti-apoptotic pathways [38]. Furthermore, TAMs play a negative role in ICT such as by preventing cytotoxic T cells from reaching tumour cells [39] as well as by promoting antibody clearance activity through Fc-Fcγ receptor binding [40]. For all these reasons, emerging macrophage-related therapeutic approaches aiming to deplete and/or shift macrophage polarisation are now promising therapeutic strategies for cancer patients.

MDSCs, a heterogeneous population of inflammation-biased monocytic (M-MDSCs) and polymorphonuclear (PMN-MDSCs) cells with immune suppressive features, share with TAMs several pro-tumour functions [23]. Whether MDSCs have a different ontogeny or represent an alternative polarisation/differentiation status of monocytes and neutrophils is still debated, yet, M- and PMN- MDSCs share many surface antigens with monocytes and neutrophils, respectively. Nonetheless, some specific markers have been recently recognised. For example, human PMN-MDSCs expressing lectin-type oxidised LDL receptor 1 (Lox1) are encountered in both blood and tumours of cancer patients and their presence is associated with worse clinical outcome [41, 42]. Moreover, CD84 protein has been identified as a specific PMN-MDSC marker in both genetically engineered mouse models (GEMM) and breast cancer patients [43]. Indeed, many investigators reported the accumulation in the PBMC fraction of low-density neutrophils (LDN) expressing CD15 and CD66b (markers shared between PMNs and PMN-MDSCs) and endowed with immune suppressive features, reminiscent of PMN-MDSCs, in individuals affected by different diseases, as cancer [44], bacterial sepsis [45], and COVID-19 [46, 47]. Despite being associated with diseases, PMN-MDSCs can also increase under physiological conditions (e.g. pregnancy [48]) or pharmacological treatments [49]. We demonstrated that both the expansion and the immunosuppressive function of MDSCs are abrogated in the absence of CCAAT/enhancer binding protein (c/EBP) β, in tumour-bearing mice, confirming the crucial role of this transcriptional factor in tumour-reprogrammed myeloid cells [50]. Notably, macrophages that are differentiated from M-MDSCs, but not from monocytes, are immune suppressive showing a restricted M-MDSC-associated genomic profile and characterised by the persistent expression of S100A9 [51]. Finally, immunosuppressive functions of MDSCs and TAMs in TME are also enforced by environmental metabolic switches such as nonderepressible-2 kinase (GCN2), confirming the intrinsic plasticity of these myeloid cell subsets [52]. In humans, M-MDSCs can be distinguished from monocytes based on low expression levels of HLA-DR [53] and activation of signal transducer and activator of transcription 3 (STAT3)-dependent signalling pathway [54, 55]. M-MDSC generation, differentiation, and function are strictly controlled by several signalling pathways which are controlled by key transcriptional factors as c/EBPβ, STAT3, and nuclear factor kappa-light chain enhancer of activated B cell (NF-κB). Recently, the compromised translocation of NF-κB p50 protein was reported to arrest the release of protein acidic and rich in cysteine (SPARC), thus abrogating reactive oxygen species (ROS)-dependent MDSC-associated immunosuppression. Indeed, the blockade of p50 translocation into the nucleus impairs the generation of immunosuppressive p50:p50 homodimers in favour of the p65:p50 inflammatory heterodimers [56]. The critical role of NF-κB p50 protein in driving MDSC differentiation has been also confirmed by the nuclear translocation of a protein complex formed by p50 in association with cellular FLICE (FADD-like IL-1β-converting enzyme)-inhibitory proteins (c-FLIP) [57]. Indeed, c-FLIP in tumour-reprogrammed monocytes does not only act as anti-apoptotic protein but also drives a marked regulation of genes encoding for immunosuppression-associated factors, like PD-L1, PD-L2, and IL-10 [57, 58]. On the other hand, aberrant FLIP expression in monocytes orchestrates pro-inflammatory pathways, leading to an unusual cytokine production fuelling massive cytokine release, feature of the cytokine release syndrome (CRS) [59]. Conversely, FLIP genetic deletion completely abrogates the generation of M-MDSCs [60], highlighting FLIP as a key regulator of this cell subset. These findings point to FLIP as a key functional-fate determinant of MDSCs, and as a novel targeting candidate to control cancer-associated immune dysfunctions.

Last-generation technologies (such as sc-RNA-seq and CYTOF) dramatically improved our current understanding of myeloid cell ontogeny and functional polarisation in cancer [61, 62] and other pathologies. To solve the myeloid-cell puzzle, Sanin and colleagues established a predictive macrophage activation model across 12 tissues and 25 biological conditions, in mice, and identified shared and unique functional pathways [63]. Moreover, TME deconvolution of about 210 patients across 15 human cancers identified many different subsets of TAMs with mixed M1/M2 signatures [64]. By taking advantage of pro-inflammatory and pro-angiogenic molecular signatures to address functional properties of different TAM subsets, the authors identified interferon-stimulated gene 15 (ISG15) and secreted phosphoprotein 1 (SPP1) gene signatures as TAMs prototypes of anti-tumour and pro-tumour macrophages, respectively. Notably, (SPP1)^+/−^ cells synergise with tumour-specific fibroblast activation protein (FAP)^+^ fibroblasts to establish a desmoplastic reaction, which limits the infiltration of cytotoxic T cells, thus restricting efficacy of checkpoint inhibitor therapy [65]. A similar macrophage’s heterogeneity has been identified in mouse models of colorectal cancer. In this context, the antibody-dependent blockade of the colony-stimulating factor-1 receptor (CSF1R) depleted TAMs with an inflammatory signature, sparing the pro-angiogenic ones [66], suggesting the use of alternative therapeutic approaches that re-educate in spite of deplete TAMs [67]. Taking together, newly available high-throughput datasets can be interrogated to expand the understanding of myeloid cell biology and define their contribution to tumour progression. Implementation of single-cell omic technology to both GEMMs and human specimens represent the new avenue to evaluate new approaches re-educating TIMs towards an anti-tumour role and for predicting patient response to therapy.

## Targeting tumour-reprogrammed myeloid cells

**Fig. 1:**
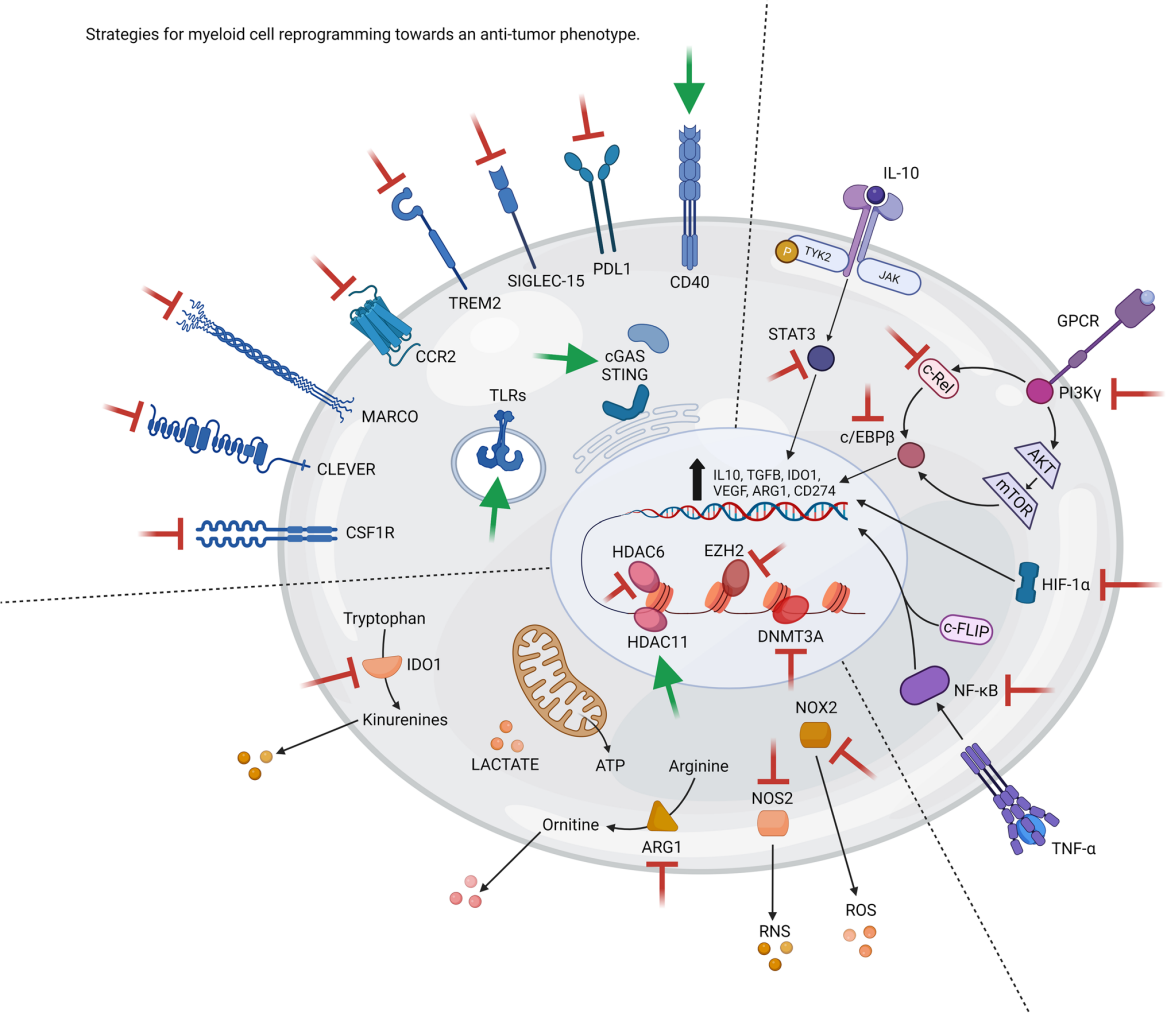
Strategies for myeloid cell reprogramming towards an anti-tumor phenotype. Myeloid cells are reprogrammed at epigenetic, transcriptional, and functional levels by tumor cells to support cancer outgrowth. Use of specific inhibitors (red) can deplete them, or avoid their recruitment in tumor. Alternatively, blocking immune suppressive switches (red Ͱ) and activating (green arrow) pro-inflammatory sensors can re-educate myeloid cells to support anti-tumor immunity. ARG1—arginase 1, ATP—adenosine triphosphate, c-FLIP—cellular FLICE (FADD-like IL-1β-converting enzyme)-inhibitory protein, cGAS—cGAMP synthase, CLEVER—common lymphatic endothelial and vascular endothelial receptor, CCR2—C–C motif chemokine receptor 2, cEBP-β—CCAAT/enhancer-binding protein beta, CSF1R—colony-stimulating factor 1 receptor, DNMT3A—DNA cytosine-5-methyltransferase 3, EZH2—enhancer of zeste homolog 2, GPCR—G protein-coupled receptors, HIF—hypoxia-inducible factor, HDAC—histone deacetylase, IDO1—indoleamine 2,3-dioxygenase 1, IL—interleukin, JAK—Janus kinase, MARCO—macrophage receptor, mTOR—mammalian target of rapamycin, NF-κB—nuclear factor kappa-light chain enhancer of activated B cells, NOS2—nitric oxide synthase 2, NOX2—NADPH oxidase, PI3K—phosphoinositide 3-kinases, RNS—reactive nitrogen species, ROS—reactive oxygen species, PDL1—programmed death-ligand 1, SIGLEC—the sialic acid-binding immunoglobulin-like lectin, STAT—signal transducers and activator of transcription, STING—stimulator of interferon genes, TGF—transforming growth factor, TLR – toll-like receptor, TNF—tumor necrosis factor, TREM—triggering receptor expressed on myeloid cells, TYK—tyrosine protein kinase, VEGF—Vascular endothelial growth factor

Compelling evidence of immune system role in tumour evolution revolutionised the development of therapies supporting immune system education to achieve a long-term control of tumour and eventually its complete debulking, other than targeting directly tumour cells. Improving immunological performances against cancer can be achieved by targeting immune-checkpoint inhibitors on adaptive immune cells for sustaining their survival and functions but also selective modulators on innate cells able to support efficiently T cell activation and limit their immunosuppressive functions. Developing effective myeloid cell-targeted approaches is a challenging research field since myeloid cells are elusive elements able to modulate their metabolism, cell-surface marker expression, and release a variety of soluble factors; therefore, the most effective strategy must aim to efficiently modulating myeloid cells’ plastic nature. Here we summarise recent findings of key players orchestrating myeloid cell biology which can be exploited to turn foes in friends (Fig. 1).

### Targeting MDSC-dependent signalling pathways

Signalling and chronic-tumour dependent hematopoietic stimulation through the release of CSF-2, CSF-3, IL-6, and other cytokines are associated with the activation of STAT3 signalling and expression of c/EBPβ transcription factor [68] which sustain MDSC expansion and immune suppressive features [50]. In accordance, c-Rel-dependent c/EBPβ upregulation orchestrates the generation of MDSCs with potent pro-tumour features [69]. Akt/mTOR-dependent activation of phosphoinositol 3 kinase pathway (PI3Kγ) triggers through the expression of c/EBPβ an immunosuppressive transcriptional program in myeloid cells supporting tumour progression [70]. Tumour-biased monocytes upregulate c-FLIP, which in turn activates an immune suppressive program, partially by NF-κB activation, including IL-10, IDO-1, and PD-L1 expression [57]. Thus, targeting of either STAT3, or c-Rel, or PI3Kγ reprogram myeloid cells towards an anti-tumour phenotype resulting in the sculpting of a TME towards the support of cytotoxic T cell response and restricting tumour progression in preclinical models of solid cancers [69–73]. Notably, some of these targeting strategies will enter soon the clinical phase, alone, or in combination with immunotherapy (Table 1). Given the antithetical role of c/EBPα (pro-inflammatory) and c/EBPβ (anti-inflammatory), and preclinical data supporting the hypothesis of c/EBPα triggering could empower anti-tumour immunity, MTL-CEBPA (an RNA-based agonist of c/EBPα) has been recently proposed in combination with anti-PD1 for the treatments of solid tumours (Table 1). However, given the broad cellular expression of several pathways and their role for physiologic regulation of tissue homeostasis and body functions, on-target and pathway-related side effects must be taken into account. c/EBP homologous protein (CHOP), which is an apoptosis-related transcription factor induced by endoplasmic reticulum (ER) stress, is essential for MDSC’s immune regulatory function [74]. CHOP expression in MDSCs was induced by tumour-linked ROS and RNS and it was regulated by the activating-transcription factor-4 (ATF4). CHOP-deficient MDSCs displayed reduced signalling through c/EBPβ, leading to a decreased production of IL-6 and low expression of phospho-STAT3 [74]. Therefore, these data highlight Chop/c/EBPβ axes as main driver of tumour-induced tolerance and targeting CHOP might represent a new valuable way to improve current cancer immunotherapies. STAT3 phosphorylated by the Janus-activated kinase (JAK) family is considered a hallmark of MDSCs [14]. STAT3 prevents MDSC apoptosis and promotes their expansion by mediating the expression of apoptosis inhibitors, including Bcl-XL, cyclin D, and cMyc. In addition, activation of STAT3 drives the production of the calcium-binding inflammatory protein, S100A8/9, and increases the accumulation of MDSCs by limiting DC differentiation and expansion [75]. ARG1 is also a downstream target of STAT3 in circulating and infiltrating MDSCs. Similarly, NADPH oxidase 2 (NOX2), induced by STAT3 in MDSCs, generates ROS that can prevent DC differentiation and antigen presentation. Finally, STAT3 blocks myeloid cell differentiation by downregulating the expression of IRF8, a transcription factor driving the development of monocytes and DCs whilst limiting granulocyte development [76]. As shown by genetic studies in mice, IRF8 inhibition is indeed responsible for halting of the PMN-MDSC differentiation and expansion [77]. Conversely, a decrease in STAT3 signalling can enable MDSC differentiation into TAMs, which often becomes the dominant tumour-infiltrating myeloid cell population [78]. Therefore, targeting STAT3 is an attractive strategy to alleviate MDSC-mediated immunosuppression in the tumour microenvironment without the need for myeloid cell depletion.

**Table 1:** Clinical trial targeting myeloid cell transcription factors, signalling pathways, epigenetic, and recruitment in association with ICT

NCT	Phase	Drug	Target	Tumor and stage	ICT
NCT04105335	I	MTL-CEBPA	Transcription factors and signaling pathways	Solid tumor	Pembrolizumab
NCT03980041	II	IPI-549 (PI3Kγ)		Solid tumors, advanced cancer	Nivolumab
NCT03961698	II	IPI-549 (PI3Kγ)		BC, RCC	Atezolizumab
NCT03334617	II	AZD9150 (STAT3)		NSCLC	Durvalumab
NCT02983578	II	AZD9150 (STAT3)		II–IV PDAC, CRC, NSCLC	Durvalumab
NCT02499328	I/II	AZD9150 (STAT3)		Advanced tumors and metastatic HNSCC	Tremelimumab
NCT03647839	II	BBI608 (STAT3)		MSI stable unresectable CRC	Nivolumab
NCT03250273	II	Entinostat	Epigenetic	Unresectable cholangiocarcinoma and pancreatic cancer	Nivolumab
NCT03765229	II	Entinostat		Melanoma	Pembrolizumab
NCT04631029	I	Entinostat		Malignant solid tumors	Atezolizumab
NCT03552380	II	Entinostat		RCC	Nivolumab, ipilimumab
NCT03978624	II	Entinostat		Bladder	Pembrolizumab
NCT01928576	II	Entinostat, azacitidine		NSCLC	Nivolumab
NCT04123379	II	BMS-813160, BMS-986253 (CCR2-CCR5, IL8)	Recruitment	NSCLC, HCC	Nivolumab
NCT03184870	I/II	BMS-813160 (CCR2-CCR5)		CRC, PDAC	Nivolumab
NCT03767582	I/II	BMS-813160 (CCR2-CCR5)		PDAC	GVAX + nivolumab
NCT03496662	I/II	BMS-813160 (CCR2-CCR5)		PDAC	Nivolumab
NCT02996110	II	BMS-813160 (CCR2-CCR5)		Advanced tumors	Nivolumab
NCT03631407	II	Vicriviroc (CCR5)		CRC	Pembrolizumab
NCT04721301	I	Maraviroc (CCR5)		CRC, PDAC	Nivolumab, ipilimumab
NCT03274804	I	Maraviroc (CCR5)		Metastatic MSS CRC	Pembrolizumab
NCT04574583	I/II	SX-682 (CXCR1-CXCR2)		Metastatic cancer, solid tumors	Bintrafusp alfa, M7824 (TGFβ PD-L1), CV301 TRICOM
NCT04599140	I/II	SX-682 (CXCR1-CXCR2)		III/IV and unresectable CRC	Nivolumab
NCT04477343	I	SX-682 (CXCR1-CXCR2)		IV PDAC	Nivolumab
NCT03161431	I	SX-682 (CXCR1-CXCR2)		III/IV melanoma	Pembrolizumab
NCT03473925	II	Navarixin (CXCR1-CXCR2)		NSCLC, CRC, prostate cancer, solid tumors	Pembrolizumab
NCT02499328	I/II	AZD5069 (CXCR2)		Advanced solid tumors, metastatic HNSCC	Tremelimumab
NCT03689699	I/II	BMS-986253 (IL8)		Prostate cancer	Nivolumab
NCT03400332	I/II	BMS-986253 (IL8)		Cancer, melanoma	Nivolumab, ipilimumab
NCT04572451	I	BMS-986253 (IL8)		Melanoma, RCC, unresectable solid tumors	Nivolumab
NCT02451982	II	BMS-986253 (IL8)		PDAC	Nivolumab, urelumab, GVAX
NCT04848116	II	BMS-986253 (IL8)		HNSCC	Nivolumab, cabiralizumab
NCT04050462	II	BMS-986253 (IL8)		HCC	Nivolumab, cabiralizumab
NCT03599362	II	Cabiralizumab (CSF1R)		PDAC	Nivolumab
NCT04331067	I/II	Cabiralizumab (CSF1R)		TNBC	Nivolumab
NCT03336216	II	Cabiralizumab (CSF1R)		PDAC	Nivolumab
NCT03158272	I	Cabiralizumab (CSF1R)		Advanced tumors	Nivolumab
NCT03502330	I	Cabiralizumab (CSF1R)		Advanced melanoma, RCC, NSCLC	Nivolumab, APX005M
NCT02452424	I/II	Pexidartinib (CSF1R)		Melanoma, NSCLC, HNSCC, GIST, ovarian cancer	Pembrolizumab
NCT02777710	I	Pexidartinib (CSF1R)		CRC, PDAC, advanced and metastatic tumors	Durvalumab
NCT03886311	II	Trabectedin (CSF1R)		Sarcoma	Nivolumab
NCT03138161	I/II	Trabectedin (CSF1R)		Advanced and metastatic sarcoma	Nivolumab, ipilimumab
NCT03085225	I	Trabectedin (CSF1R)		Ovarian cancer and soft tissue sarcoma	Durvalumab
NCT03590210	II	Trabectedin (CSF1R)		Metastatic soft tissue sarcoma	Nivolumab
NCT02323191	I	Emactuzumab (CSF1R)		Solid cancer	Atezolizumab

### Targeting epigenetic modifications

DNA methylation and histone acetylation/deacetylation catalysed, respectively, by DNA methyl-transferases (DNMTs) and histone acetyltransferases (HATs) and deacetylases (HDACs) are often altered in tumour-reprogrammed myeloid cells. Indeed, many epigenetic mechanisms influence MDSC differentiation and functions, thus remodelling TME [79]. Hence, a pharmacological targeting of epigenetic modifiers can be potentially considered for combinatorial immunotherapy approaches aiming at both triggering an immuno-mediated tumour cell recognition and, simultaneously, reducing MDSC-dependent immune suppression. EZH2 inhibition, a histone-lysine N-methyltransferase, has reported to suppress anti-tumour immunity by driving MDSC differentiation from primitive hematopoietic progenitors reducing CD4^+^ and IFNγ^+^CD8^+^ T cells in lung and colon carcinoma models [80]. On the other hand, a pan-HDAC inhibition decreased MDSC accumulation in a mammary tumour model by promoting the apoptosis of MDSC-differentiating Gr-1^+^ cells. This selective depletion was partially due to increased intra-cellular reactive oxygen species that, consequently, was associated to an increased proportion of IFNγ- or perforin-producing CD8^+^ T cells [81]. Recently, histone (de)acetylation has shown to be involved in the polarisation of M-MDSCs in PMN-MDSCs [82]. In detail, *Youn and colleagues* have reported that a large proportion of M-MDSCs in tumour-bearing mice could acquire phenotypic, morphological, and functional features of PMN-MDSCs, rather than DCs or macrophages. This process is governed by epigenetic transcriptional silencing of the retinoblastoma gene controlled by HDAC-2. HDAC inhibition re-directs M-MDSC differentiation towards macrophages and DCs [82]. Furthermore, HDAC11 has emerged as a negative regulator of MDSC expansion and function in a lymphoma model by controlling IL-10 gene expression [83]. Indeed, immature myeloid cells to MDSC transition require a decreased expression of HDAC11 [83]. Conversely, the bromodomain of the HAT CBP/EP300 is a critical regulator of H3K27 acetylation in MDSC promoters and enhancers of pro-tumourigenic genes [84]. CBP/EP300-BRD inhibition redirects tumour-associated MDSCs from a suppressive to an inflammatory phenotype through STAT pathway-related gene downregulation and ARG1 and iNOS inhibition, thus limiting tumour growth in a model of colon carcinoma [84]. Interestingly, the epigenetic-associated component p66a, a subunit of the Mi2/NuRD HDAC complex, has shown to suppress STAT3 phosphorylation and ubiquitination by directly interacting with STAT3 protein, providing novel insights in controlling STAT3 activation in myeloid cell differentiation and activation [85]. On the other hand, class I HDAC inhibition has been reported to support anti-tumour response by dampening ARG-1, iNOS, and COX-2 levels in MDSCs as well as altering the release of cytokines/chemokines [86]. Interestingly, the inhibition of class I HDAC with entinostat selectively reduces the immunosuppressive activity mediated by PMN-MDSC, without any effect on M-MDSCs or macrophages [87]. Indeed, M-MDSC displayed higher levels of class II HDAC6, and its inhibition with ricolinostat reduces M-MDSC suppressive activity, without affecting PMN-MDSCs. However, only the combination of both molecules impacts on tumour progression [87].

MDSC properties are also influenced by specific changes in DNA methylation patterns. For instance, Durkin and colleagues demonstrated for the first time the ability of the demethylating agent decitabine to promote the differentiation of tumour-infiltrated CD11b^+^ cells into mature F4/80/CD11c/MHC class II-positive APCs [88]. Decitabine strongly reduces the release of immune suppressive and pro-inflammatory factors by tumour-derived myeloid cells, and, overall, naïve mice receiving ex vivo reprogrammed tumour-derived myeloid cells were protected from tumour outgrowth [88]. More recently, the downregulation of DNMT3A deletes MDSC-specific hypermethylation and abrogates their immunosuppressive capacity [89]. Ovarian cancer patient-derived MDSCs showed a similar hypermethylation signature in association with a prostaglandin E2 (PGE2)-dependent DNMT3A overexpression [89]. Furthermore, decitabine employment reduces tumour cell proliferation and trigger T cell immune response by depleting M-MDSCs in different tumour models [90, 91]. Taken together, these data suggest the control of DNA methylation as promising scenario for clinical approach (Table 1). On the other hand, the use of combinatorial epigenetic drugs not only could have an impact on tumour progression, but also might prevent the formation of the premetastatic niche [92]. In accordance, adjuvant epigenetic therapy with low-dose DNMT and HDAC inhibitors disrupts the premetastatic niche by blocking the trafficking of MDSCs through the downregulation of CCR2 and CXCR2 and also favouring MDSC differentiation into anti-tumour macrophage-like cells [92]. Collectively, these findings clearly reveal epigenetic drugs as potent tumour-reprogrammed myeloid cell-targeting agents that could be used to enhance the efficacy of ICT.

### Targeting myeloid-cell recruitment networks

The recruitment of M-MDSCs is mainly mediated by tumour-expressing C–C motif chemokine ligand 2 (CCL2) in several tumour types, including breast, ovarian, gastric, and colorectal cancer [93]. Indeed, tumour-associated myeloid cells from patients frequently express CCR2 [94, 95]. Hence, blocking the CCL2-CCR2 interaction could be an effective therapeutic approach to prevent the accumulation of pro-tumour myeloid cells within TME. Promising results have been reported in several preclinical tumour models [96–99]. The seminal work by Pollard’s laboratory demonstrated that the therapeutic blockade of CCL2-CCR2 axis interrupts the recruitment of inflammatory monocytes and inhibits metastasis in vivo, prolonging the survival of mice bearing breast cancer [95]. However, the interruption of CCL2 inhibition has shown to promote a rebound of pro-tumour myeloid cells inducing mouse breast cancer metastasis [100]. Furthermore, prostate cancer patients enrolled in a phase II clinical trial using an anti-CCL2 monoclonal antibody showed an increased CCL2 release following anti-CCL2 interruption [101], suggesting that a continuous arrest of CCL2 is mandatory to control tumour progression. Several other cytokines and chemokine receptors have been reported to induce monocyte and neutrophil recruitment, like CCL5 and CCL7 [102, 103] and CCL3 [104]. Recently, a therapeutic approach by in vivo silencing of CCR1 and CCR5 on myeloid cells has shown to strongly inhibit tumour progression by converting PMN-MDSCs into anti-tumour neutrophils [105]. An interesting report showed that metastatic tumours often overexpressed CSF-3, leading to the expansion and mobilisation of Ly6G^+^Ly6C^+^ granulocytes, which in turn produced Bv8, a protein implicated in angiogenesis and mobilisation of myeloid cells. This process creates a positive feedback loop with the consequent accumulation of PMNs in organ-specific metastatic sites resulting in an increased metastatic ability [106]. Of note, recent studies suggest that CD200-CD200 receptor (CD200R) interaction might be essential in controlling the myeloid heterogeneity in tumours by a mechanism involving CD200-expressing endothelial cells [107, 108]. In addition, the expression of CD200 was found in human pancreatic cell lines and CD200R expression was found at high level on PDAC patient-derived MDSCs [108]. In vivo studies demonstrate that CD200 antibody blockade limits the percentage of tumour-infiltrating MDSCs, but the significance and the mechanisms underlying CD200-CD200R interaction in tumour microenvironment remain to be clarified [108].

### Targeting MDSC-released cytokine and immune mediators

Secretion of soluble factors able to fuel a pathological inflammation or inhibit immunological responses in TME is considered a key mechanistic pathway of MDSC’s biology. Here we summarised the most relevant MDSC-released cytokines/growth factors able to sustain tumour progression.

Emergency myelopoiesis in BM is influenced by persistent stimulation from tumors. These signals include cytokines, namely, CSF-1, -2, and -3, whose function is often overlapped and converging to signaling pathways of the JAK/STAT/ERK/PI3K axes [112]. In this contest, it was shown that mouse breast 4T1 cancer cells release CSF-2 and CSF-1, promoting BM output of MDSCs and preparing the premetastatic niche [106]. Once in the lung, MDSCs induce angiogenesis and sustain the metastatic spread by, in turn, releasing CSF-2. Chemotherapy significantly enhanced the production of CSF-2 from various tumors (e.g. pancreatic adenocarcinoma, PDAC) [113]. Further, CSF-2 and CSF-3 were shown to induce the differentiation of monocytes into MDSCs and to stimulate myelopoiesis in BM of in mouse model of PDAC and glioblastoma [114].

The cytokine IL-6 was found to be a crucial regulator of MDSC accumulation, activation, and differentiation in vitro as well as a factor promoting tumour cell proliferation, survival, invasiveness, and metastasis. Accordingly, IL-6 can be used as a negative prognostic marker in cancer. Within the tumour microenvironment and in the periphery, IL-6 promotes differentiation of myeloid precursors into MDSCs and reinforces their suppressive function by promoting and maintaining STAT3 phosphorylation [115]. For example, phosphorylated STAT3 levels in MDSCs isolated from head and neck squamous cell carcinoma (HNSCC) patients positively correlate with ARG1 expression and suppression of autologous T cell proliferation [54]. Since STAT3 is a downstream regulator of IL-6, which was found to be associated with a worse survival in HNSCC patients [116], a correlation between IL-6 levels, STAT3 phosphorylation, and ARG1 expression could be proposed.

The cytokine IL-1β drives MDSC expansion and migration [117]. IL-1β concentration was positively correlated with M-MDSC subset [118] in the peripheral blood of advanced melanoma patients. Moreover, M-MDSC produces IL-1β, which in turn upregulates E-selectin expression, favouring tumour cell arrest on endothelial cells, in preclinical cancer models [119]. In other studies, tumour-derived NLRP3 increases the expression and secretion of IL-1β by MDSCs [120]. Currently, several agents are available to inhibit IL-1β, including IL-1Ra (anakinra), IL-1β-specific antibodies (canakinumab), and inflammasome inhibitors [121, 122]. Notably, multiple cancer therapeutic agents such as chemotherapeutic drugs, MAPK inhibitors, and BRAF V600E inhibitor (BRAFi) have been reported to either increase the expression of IL-1β or activate inflammasomes in myeloid cells causing unwanted side effects. In this regard, IL-1β blockade may generate adjunctive effects when combined with chemotherapies or other treatments in cancer.

TGF-β is a well-documented immunosuppressive cytokine secreted by MDSCs in tumour-bearing host [123]. How MDSC-derived TGF-β is released and regulated remains elusive. It was shown that MDSC-derived TGF-β is induced by IL-13 and CD1d-restricted T cells, most likely natural killer T (NKT) cells, in vivo [124]. Recent studies have shown that TGF-β production by MDSCs is regulated by TNF-α and semaphorin 4D, in vitro [125, 126]. In contrast, CD14^+^HLA-DR^−/low^ MDSCs from patients with liver cancer show no TGF-β secretion [127], suggesting that TGF-β production by MDSCs may be context-dependent. MDSC-derived TGF-β contributes to T cell suppression. Song et al. have shown that tumour-derived MDSC transfer to asthmatic mice leads to reduced pulmonary recruitment of inflammatory cells, suppressed Th2 response, and decreased IgE production in a TGF-β1-dependent manner [128]. Furthermore, TGF-β is essential in Treg induction by MDSCs. In addition to immune suppression, TGF-β has been implicated in the regulation of tumour metastasis facilitated by MDSCs. A portion of tumour cells undergoes epithelial-mesenchymal transition (EMT) to disseminate, invade surrounding tissue, and metastasize. In a spontaneous murine model of melanoma, *Toh and colleagues* have shown for the first time that MDSCs use TGF-β, epidermal growth factor, and hepatocyte growth factor to induce EMT and to support metastasis and that MDSC depletion reverts this phenotype [125], confirming TGF-β as a critical target to abolish MDSC’s pro-tumour features.

Tumour-reprogrammed myeloid cells also actively influence angiogenesis, which represents a hallmark of cancer progression [17]. Anti-angiogenic therapies targeting vascular-endothelial growth factor (VEGF) and cognate receptors provided clinical benefits in different kind of solid tumours (including colorectal, kidney, lung, ovarian, brain tumours) [129], although often, response to therapy was limited in time and in a fraction of treated patients. In part this is mediated by compensatory circuits of sustained angiogenesis mediated by tumour-infiltrating myeloid cells [130–132]. In accordance, single-cell-mediated TIM deconvolution underscores the association of pro-angiogenic TAMs with worse overall survival in different solid tumour contexts [64]. Thus, repolarisation of TIMs from pro-angiogenic to anti-tumour phenotype can synergise with anti-angiogenic therapy to support long-term control of tumour progression. Clinical trials targeting VEGF and other soluble mediators are listed in Table 2.

**Table 2:** Clinical trial targeting myeloid cell soluble mediators in association with ICT

NCT	Phase	Drug	Target	Tumor and stage	ICT
NCT05180006	II	Bevacizumab (VEGFR)	Soluble mediators	BC	Atezolizumab
NCT02997228	III	Bevacizumab (VEGFR)		Metastatic CRC	Atezolizumab
NCT04262687	II	Bevacizumab (VEGFR)		Metastatic CRC	Pembrolizumab
NCT04524871	I/II	Bevacizumab (VEGFR)		HCC	Atezolizumab, tocilizumab
NCT03434379	III	Bevacizumab (VEGFR)		Unresectable HCC	Atezolizumab
NCT03955354	II	Apatinib (VEGFR)		Melanoma	SHR1210 (PD1)
NCT04691817	I/II	Tocilizumab (IL6R)		NSCLC	Atezolizumab
NCT04258150	II	Tocilizumab (IL6R)		PDAC	Nivolumab, ipilimumab
NCT03821246	II	Tocilizumab (IL6R)		Prostate cancer	Atezolizumab
NCT04940299	II	Tocilizumab (IL6R)		III/IV solid tumors	Nivolumab, ipilimumab
NCT03012230	I	Ruxolitinib (JAK)		TNBC, metastatic stage IV BC	Pembrolizumab
NCT03026140	II	Celecoxib (COX-2)		Colon carcinoma	Nivolumab, ipilimumab
NCT03728179	I	Celecoxib (COX-2)		Solid tumors	Nivolumab Ipilimumab
NCT03926338	I/II	Celecoxib (COX-2)		CRC MSI-H	Toripalimab
NCT02959437	I/II	Epacadostat (IDO-1)		Advanced and metastatic tumors	Pembrolizumab
NCT03006302	II	Epacadostat (IDO-1)		Metastatic PDAC	Pembrolizumab GVAX
NCT03463161	II	Epacadostat (IDO-1)		HNSCC	Pembrolizumab
NCT02752074	III	Epacadostat (IDO-1)		Melanoma	Pembrolizumab
NCT04200963	I	IK-175 (AhR)		Locally advanced or metastatic solid tumors and urothelial carcinoma	Nivolumab
NCT02903914	I/II	INCB001158 (ARG1)		Advanced/metastatic solid tumors	Pembrolizumab
NCT03361228	I/II	INCB001158 (ARG1)		Advanced solid tumors	Pembrolizumab
NCT03236935	I	L-NMMA (NOS)		NSLC, HNSCC, IV melanoma, bladder carcinoma, Hodgkin’s lymphoma	Pembrolizumab
NCT04265534	II	CB-839 (glutaminase)		NSLC	Pembrolizumab

### Targeting metabolic pathways related to immune suppression

Myeloid cells can affect T cell recruitment and activity in TME by employing an arsenal of soluble mediators, including metabolites (i.e. NO, ROS, RNS, adenosine, α-ketoglutarate, prostaglandin, kynurenine, lactate) able to alter T cell function and survival [133, 134]. MDSCs sense and adapt to nutrient changes by acquiring the most effective pathways to maintain their immunosuppressive and pro-tumour functions. We detail below the most recent updates on major metabolic pathways.

#### Amino acid

Increased amino acid metabolism in MDSCs impairs anti-tumour T cell activity [135]. Especially, arginine, glutamine, and tryptophan have been shown to control viability, polarisation, and motility as well as effector function of anti-tumour T cells [42, 136]. Increased consumption of L-arginine by ARG1 is one of the known immunosuppressive mechanisms set in place by MDSCs to enhance tumour-immune escape. In fact, depletion of arginine down-modulates the expression of CD3ζ chain and alters GCN2 in T cells, resulting in TCR loss of function and cell cycle arrest, respectively [137–139]. Alongside, products derived from ARG1 metabolism, as ornithine, putrescine, and spermidine broadly promote MDSC immunosuppressive function, particularly in brain tumours [140, 141]. iNOS also contributes to fine tune the amount of L-arginine in the TME. The metabolic product derived from L-arginine degradation, NO, is known to increase T cell apoptosis by impairing IL-2R signalling trough JAK-3, STAT5, ERK, and Akt [142, 143]. In fact, NO directly increases DNA damage response, mitochondrial ROS generation, and produces peroxynitrites (PNT) in a NADPH-dependent manner, by reacting with superoxide anion [144, 145]. In addition, PNT favours post-translational modification of specific chemokines and cytokines (e.g. CCL2 and CSF-2) which have been shown to favour the recruitment and infiltration of anti-tumour T cells as well as they critically alter MHC-peptide complex inhibiting T cell activation [146–148]. Moreover, sustained type I interferon signalling exacerbate NO production by aberrant iNOS expression inducing anti-PD1 resistance [149]. On the other hand, NO produced by a subset of myeloid cells infiltrating the tumour, Tip-DCs, sustain the anti-tumour activity of adoptively transferred tumour-specific CD8^+^ T cells [150]. These data suggest that the outcome of amino acid depletion in the TME is cell dependent and future novel therapeutic strategies will have to consider.

Glutamine is one of the most abundant amino acids present in the blood. In cancer, glutamine is converted in glutamate and α-ketoglutarate, to support nucleoside and lipid biosynthesis, and to sustain protein glycosylation [151, 152]. Oncogenes, such as c-MYC and KRAS, greatly increase the uptake and catabolism of glutamine in cancer cells [153, 154], further enhancing the paucity of glutamine available in the TME for both tumour-promoting or -killing mechanisms. In MDSCs, L-glutamine fuels the tricarboxylic acid (TCA) cycle providing the intermediates and energy for the development and effector functions. Besides competing with tumour cells for glutamine, MDSCs oxidise L-glutamine in an AMPK-dependent manner, which increases and sustains their immunosuppression function [155]. In line with this observation, MDSCs were shown to increase glutamine biosynthesis and transglutaminase (TGM) activity in a murine model of metastatic mammary tumours [156] and TGM expression in MDSCs was correlated to the metastasis and multi-drug resistance of breast cancer [157].

Tryptophan catabolism is mediated in mammals by two closely related indoleamine-pyrrole 2,3 dioxygenase enzymes (indoleamine 2,3 dioxygenase [IDO]1 and IDO2) and the unrelated enzyme tryptophan 2,3 dioxygenase (TDO) [158]. IDO1 is primarily expressed by myeloid cells and stroma in response to inflammatory immune signals whereas IDO2 and TDO are largely unresponsive to immune stimuli and have a broader expression pattern [159]. Research examining the role of IDO1-mediated immune regulation has focused on the effect of amino acid consumption (i.e. amino acid starvation, stress) and the production of effector catabolites. IDO1-generated N-formyl-L-kynurenine is further catabolised by aryl formamidase to form L-kynurenine (L-Kyn). L-Kyn is a key product of IDO1 catabolism of tryptophan and, together with other downstream catabolic products (e.g. cinnabaric acid), is a regulator of immunity by direct binding to the aryl hydrocarbon receptor (AhR) [160]. AhR is a cytoplasmic receptor/transcription factor with a key role in immune function. AhR function in MDSCs has not been examined in detail. Nonetheless, AhR potently impacts hematopoietic progenitor development driving expansion of precursors [161] and AhR signalling may promote differentiation of leukemic stem cells in acute myeloid leukaemia. AhR signalling impacts MDSC expansion and differentiation by causing proliferation and differentiation of hematopoietic precursors and promoting the emergency granulopoiesis [50]. Together, these studies support the ability of AhR activation to induce highly immunosuppressive cells of the myeloid lineage. Therefore, the ability to control AhR activation is becoming imperative to modulate immunosuppression and inflammation. Recently, nanoparticles (NPs) have been engineered to re-establish tolerance via AhR activation [162]. Together, the success of these studies would provide great promise for AhR as a therapeutic for immunomodulation.

#### Lipids

Lipid metabolism regulates both differentiation, expansion, and effector function of MDSCs. High-fat diet favours the differentiation of MDSCs from BM-derived precursors and potentiates the suppressive activity of these cells, in mice [163]. In tumour-bearing mice, obesity is associated with an increased accumulation of MDSCs and a reduced CD8^+^ T cell to MDSC ratio and elevated adiposity is also associated with the accumulation of MDSCs in the spleens and lymph nodes of tumour-free mice. In MDSCs, lipids enter cells by the scavenger receptor CD36, and promote the switch from glycolysis to fatty acid oxidation (FAO), as a primary source of energy [164]. In accordance, the deletion of CD36 or FAO inhibition deprives MDSCs of their immunosuppressive function, delaying tumour growth and enhancing the efficacy of chemotherapy and immunotherapy [165]. Recently, the fatty acid transport protein 2 (FATP2) was identified as a regulator of the suppressive functions of PMN-MDSCs. FATP2 is responsible for arachidonic acid uptake and subsequent PGE2 synthesis. FATP2 inhibition abrogates PMN-MDSC-suppressive functions and enhances cancer immunotherapy efficacy [166].

#### Glucose

MDSCs rely in glycolysis, the pentose phosphate, and TCA pathways to differentiate and fulfil their functions [15]. Indeed, MDSCs are endowed with a high glucose and glutamine uptake rates, a reduced oxygen consumption rate, and most of their synthesised ATP is obtained through glycolysis-dependent mechanisms [167, 168]. Thus, high glycolytic flux is required for MDSC maturation from bone marrow precursors suggesting an indirect immune suppressive mechanism towards T cells mediated by carbon source consumption. Moreover, the upregulation of glycolytic pathways protects MDSCs from apoptosis and contributes to their survival by preventing ROS-mediated apoptosis via the anti-oxidant activity of the glycolytic intermediate phosphoenolpyruvate. Glycolysis rate is associated with sustained ARG1 activity in MDSCs. Under hypoxic conditions, HIF1α activation triggers the oxidative phosphorylation to glycolysis switch in MDSCs [169]. HIF1α is a critical differentiation and function regulator of MDSCs in the TME [170]. In fact, M-MDSCs show a dormant metabolic state, fail to metabolise glucose, and have a reduced cellular ATP content and low basal mitochondrial respiration [171]. This peculiar metabolic phenotype is regulated by methylglyoxal accumulation in MDSCs, which is then transferred to T lymphocytes. Methylglyoxal suppresses T cell function by the chemical depletion of L-arginine, as well as by rendering L-arginine-containing proteins non-functional through a glycation-dependent mechanism.

Notably, many of these immune suppressive circuits are interconnected and sustained in catalytic loops: for example, metabolic shift from glycolysis to oxidative phosphorylation in myeloid cells increases the production and release of ATP, which in turn acts on CD39, CD73, and adenosine receptors to support an immunosuppressive transcriptional program. According to this, recent clinical trials have been designed in order to evaluate the prominence and safety of specific inhibitors for many enzymes (i.e., IDO, ARG1, NOS2) alone or in combination with target therapy and checkpoint inhibitors (Table 2) [172, 173]. For example, epacadostat, that is a nanomolar inhibitor of IDO1, has been confirmed to be effective and safe when combined with immune checkpoint inhibitors in patients with melanoma (combined with ipilimumab) and those with non-small-cell lung cancer, squamous cell carcinoma of the head and neck, renal cell carcinoma, and urothelial carcinoma (combined with pembrolizumab) in phase I/II. However, a phase III study (NCT02752074) of epacadostat combined with pembrolizumab in patients with unresectable or metastatic melanoma demonstrated that this compound did not enhance anti-PD-1 therapeutic effect [174]. The unexpected results may be the consequence of improper study design, insufficient drug exposure, and/or inappropriate combination strategy. Indeed, enrolled patients were not selected according to the expression of IDO in TME. Guaranteeing sufficient drug exposure, testing new combination protocols, and performing distinctive analysis of primary and secondary endpoints in both IDO positive and negative patients’ subpopulations should be taken into consideration in the future phase III clinical trials. Furthermore, a more precise clarification of the biology of enzyme targets in TME is mandatory to understand where and when these pathways have to be targeted. For instance, IDO1 contains two functional immunoreceptor tyrosine-based inhibitory motifs (ITIMs), which modulate the immune response of IDO1-expressing myeloid cells [175–177]. Moreover, IDO1 controls also a multi-pronged anti-ferroptotic death pathway, which plays a pivotal role in tumour suppression in TME [178]. Indeed, IDO1^+^ cells export kynurenines, which are imported by non-IDO1-expressing cells via solute carrier transporters, and these tryptophan catabolites are converted in metabolites with anti-ferroptotic activity [179], supporting local immunosuppression and cancer cell proliferation. To overcome these substantial limitations linked to the pleiotropic effects of the activity of these enzymes [58], a strategy based of active vaccination can be exploited. This active immunotherapeutic strategy has been also validated in a clinical trial, in which patients with metastatic melanoma were treated with a combinatorial IDO/PD-L1-targeting approach based on IDO1-peptide vaccine combined with nivolumab [180].

### Targeting cell-to-cell interaction

Physical interactions influence a bidirectional crosstalk between myeloid cells and different TME cell components, including tumour cells, stromal cells, and immune cells. Amongst cell-to-cell interactions leading to switch off T cell-mediated anti-tumour response, immune checkpoints are crucial inhibitory pathways responsible for cancer immune evasion. MDSCs express both PD-L1, interacting with PD-1 expressed on T cells, and CTLA-4. PD-1 prevents T-cell immune-reactivity via engaging with PD-L1 expressed on tumour/myeloid cells in the “canonical” PD-1/PD-L1 axis. Whilst the effect of the canonical PD-1/PD-L1 axis and its inhibition have been extensively described [181], several evidences regarding the presence of a myeloid-dependent “non-canonical” PD1/PD-L1 function could represent a clear turning point for a reconsideration of PD-1/PD-L1 axis targeting. Indeed, different studies have confirmed PD-1 expression on monocytes, macrophages, DCs, and MDSCs in tumour models and patients [182–187]. PD-L1 could be induced on activated T cells and interact with intra-tumoural PD-1 myeloid cells, resulting in various pro-tumour effects [188]. As an example, PD-1 suppressed STAT1- and NF-κB-mediated M1 polarisation promoting M2 polarisation by increasing STAT6 phosphorylation [189, 190]. Recently, Gordon et al. demonstrate that PD-1 expression on TAMs strongly reduces the phagocytic activity against tumour cells [183]. Thus, considering the role of myeloid-PD-1 during ICT, these findings could have important therapeutic implications. The role of myeloid CTLA-4 remains partially unclear. The cis CTLA-4 blockade on T cell has been reported by different authors, but its function in mediating T cell inhibition by MDSCs is still unclear and under investigation [191]. CTLA-4 ligands, such as B7 molecules, are highly expressed by TAMs and DCs in the tumour microenvironment and their expression directly correlates with the reduction of anti-tumour T cell by inhibiting CD28 in several tumour models [192, 193]. Moreover, myeloid cells express as well inhibitory receptors promoting immune suppressive functions, including scavenger receptors as macrophage receptor with collagenous structure (MARCO) and triggering receptor expressed on myeloid cells 2 (TREM-2), common lymphatic endothelial and vascular endothelial receptor 1 (CLEVER), and Ig-like receptors, such as sialic acid-binding immunoglobulin-like lectins (SIGLEC15) and V-domain Ig suppressor of T cell activation (VISTA). These receptors are associated with anti-inflammatory immune suppressive phenotype of myeloid cells which dampens T cell activation and function [194–197]. Antibodies blocking those receptors recently entered the clinical evaluation phase alone or in combination with ICT (Table 3) and they could open a new age of cancer immunotherapy in the near future. For instance, TREM-2 is an activating receptor of the Ig superfamily that binds lipids and transduces intra-cellular signals through the adaptor DAP12 [198]. DAP12 recruits the protein tyrosine kinase Syk, which initiates a cascade of tyrosine phosphorylation events which lead to the activation of PLCγ2, PI3K, mTOR, and MAPK, ultimately leading to cell activation, metabolic adaptation, and transcriptional rearrangement [199]. TREM-2 is expressed in TAMs and MDSCs [194, 200, 201]. To support its function, a subset of myeloid cells co-expressing ARG1 and TREM-2 were identified in several preclinical models of cancers and genetic ablation of TREM-2 in mice inhibited accumulation of intra-tumour myeloid cells, leading to a decrease in dysfunctional CD8^+^ T cells and reduced tumour growth [194]. TREM-1 and TREM-2 are expressed on MDSCs and TAMs and correlate with tumour increased volume in preclinical 4T1-breast cancer model. In accordance, high TREM2 expression on tumour myeloid cells is associated with a poor survival rate in patients with colorectal carcinoma or triple-negative breast cancer [201]. Recent reports highlight a novel role for the apolipoprotein E (APOE)-TREM-2 axis in cancer [194, 202], providing promising novel therapeutic targets. TREM-2-deficiency enhances the efficacy of the anti-PD-1 treatment and antibody-dependent TREM-2 blockade is sufficient to remodel the intra-tumour myeloid compartment and to slow tumour growth. On the other hand, expression of some APOE variants, like APOE4, is associated with an improved responsiveness of melanoma patients to anti-PD1 ICT, suggesting that both APOE and TREM-2 expressions on myeloid cells can be used to stratify patients who might benefit from this therapeutic strategy.

**Table 3:** Clinical trial targeting myeloid cells receptors and cytosolic sensors in association with ICT

NCT	Phase	Drug	Target	Tumor and stage	ICT
NCT04799431	I	Poly-ICLC (TLR3)	**Receptor sensors**	Metastatic PDAC and CRC	Retifanlimab
NCT02826434	I	Poly-ICLC (TLR3)		BC	Durvalumab
NCT03721679	I/II	Poly-ICLC (TLR3)		Solid tumors	Atezolizumab, durvalumab
NCT02834052	I/II	Poly-ICLC (TLR3)		Metastatic CRC, solid tumors	Pembrolizumab
NCT02643303	I/II	Poly-ICLC (TLR3)		Solid tumors	Durvalumab, tremelimumab
NCT04508140	II	BO-112 (TLR3)		Oesophageal, gastric, colon cancer	Pembrolizumab
NCT04777708	I	BO-112 (TLR3)		Advanced refractory HCC	Pembrolizumab
NCT04420975	I	BO-112 (TLR3)		Sarcoma	Nivolumab
NCT04134000	I	BCG (TLR2-4)		Invasive bladder cancer	Atezolizumab
NCT03982121	I	GLA-SE (TLR4)		Metastatic CRC	Nivolumab, ipilimumab
NCT02609984	II	GLA-SE (TLR4)		Sarcoma	Atezolizumab
NCT02501473	I/II	GLA-SE (TLR4)		Lymphoma	Pembrolizumab, rituximab
NCT03447314	I	GSK1795091 (TLR4)		Advanced solid tumors	Pembrolizumab
NCT04072900	I	Imiquimod (TLR7)		Melanoma	Toripalimab
NCT03982004	I	Imiquimod (TLR7)		Metastatic melanoma	Pembrolizumab
NCT03276832	I	Imiquimod (TLR7)		Advanced and metastatic melanoma	Pembrolizumab
NCT04101357	I/II	BNT411 (TLR7)		Solid tumors	Atezolizumab
NCT02556463	I	MEDI9197 (TLR7-8)		Solid tumors	Durvalumab
NCT04799054	I/II	TransCon (TLR7-8)		Advanced and metastatic tumors	Pembrolizumab
NCT05081609	I/II	TransCon (TLR7-8)		Advanced and metastatic tumors	Pembrolizumab
NCT04840394	I	BDB018 (TLR7-8)		Solid tumors	Pembrolizumab
NCT03435640	I/II	NKTR-262 (TLR7-8)		TNBC, melanoma, RCC, CRC, HNSCC, sarcoma	Nivolumab
NCT04460456	I	SBT6050 (TLR8)		HER2 + tumors	Pembrolizumab
NCT04612530	I	CpG (TLR9)		Advanced and metastatic PDAC	Nivolumab
NCT03831295	I	SD101 (TLR9)		Advanced and metastatic solid tumors	OX-40
NCT03007732	II	SD101 (TLR9)		Prostatic tumors	Pembrolizumab
NCT04050085	I	SD101 (TLR9)		Metastatic PDAC	Nivolumab
NCT02521870	I/II	SD101 (TLR9)		Metastatic melanoma, HNSCC	Pembrolizumab
NCT05220722	I/II	SD101 (TLR9)		HCC, intra-hepatic cholangiocarcinoma	Pembrolizumab, nivolumab, ipilimumab
NCT04401995	II	CMP-001 (TLR9)		Melanoma	Nivolumab
NCT04708418	III	CMP-001 (TLR9)		III/IV melanoma	Pembrolizumab
NCT03326752	I	DV281 (TLR9)		NSLC	Nivolumab
NCT04220866	II	MK-1454 (STING)		HNSCC	Pembrolizumab
NCT04708418	II	GSK3745417 (STING)		Advanced metastatic melanoma	Pembrolizumab
NCT04609579	I	SNX281 (STING)		Advanced solid tumors	Pembrolizumab
NCT03249792	I	MK-2118 (STING)		Advanced metastatic tumors	Pembrolizumab
NCT03956680	I	BMS-986301 (STING)		Advanced solid tumors	Nivolumab, ipilimumab
NCT04020185	I/II	IMSA101 (STING)		Solid tumors	ICT
NCT03424005	I/II	Selicrelumab (CD40)		TNBC	Atezolizumab, tocilizumab
NCT02706353	I/II	Sotigalimab (CD40)		Melanoma	Pembrolizumab
NCT03597282	I	Sotigalimab (CD40)		Metastatic melanoma	Nivolumab, ipilimumab
NCT03214250	I/II	Sotigalimab (CD40)		Metastatic PDAC	Nivolumab
NCT04993677	II	SEA-CD40 (CD40)		Melanoma NSLC	Pembrolizumab
NCT01103635	I	CP-870893 (CD40)		Recurrent/IV melanoma	Tremelimumab
NCT04886271	II	HX009 (CD47/PD1 bispecific antibody)		Advanced solid tumors	
NCT02518958	I	RRx-001 (CD47)		Malignant solid tumors	Nivolumab
NCT03558139	I	Magrolimab (CD47)		Ovarian cancer	Avelumab
NCT04060342	I/II	GB1275 (CD11b)		Advanced solid tumors	Pembrolizumab

### Targeting pro-tumour features by myeloid cell-reprogramming

Myeloid cells play a critical role and promptly respond to infections and danger signals and in supporting activation of adaptive immune response towards foreign antigens and pathological conditions (cancer included). In order to finely tune immune system activation and relief, myeloid cells are endowed with both activation and inhibition receptors. Pattern recognition receptors (PRR), such as toll-like receptors (TLR), nod-Like receptors (NLRs), and cytosolic sensors such as stimulator of interferon genes (STING) involved in the sensing of pathogen-associated molecular patterns (PAMPs) and danger-associated molecular patterns (DAMPs), are responsible for tuning both myeloid activation and further polarisation of immune response towards an anti-tumour or pro-tumour phenotype [203]. In accordance, many TLRs agonists (imiquimod for TLR7 and CpG for TLR9) or cGAS-STING triggers were employed to activate anti-viral interferon-mediated immune responses with final aim to polarise myeloid cells towards an anti-tumour state, prime, and support T cell-mediated anti-tumour immunity [204, 205]. For instance, monophosphoryl lipid A-mediated TLR4 triggering synergises with IFN-γ to activate a pro-inflammatory type 1 IFN conversion of macrophages isolated from metastatic pleural effusions of breast cancer patients conferring them direct anti-tumour cytotoxic abilities. Moreover, intra-peritoneal or intra-tumour injection of reprogrammed macrophages controls tumour progression and metastatic spreading of breast and ovarian tumours, in preclinical models [206]. Notably, the adoption of PRR agonists can support the infiltration of T lymphocytes in cold tumours, such as pancreatic cancer [204, 207], and synergise with ICT to revert immunotherapy resistance in different preclinical models of solid cancers [208, 209]. Another, non-redundant (PRR independent) strategy to activate myeloid cells to directly reshape TME contexture and sustain a tumour-specific T cell immune response is mediated by CD40 triggering, which is able to support immune response in tumours resistant to immunotherapy such as PDAC, in both mouse models and human patients [210, 211]. These data pave the way to phase 1 clinical evaluation of CD40 agonist with chemotherapy and ICT combination in pancreatic cancer patients which showed clinical activity and deserve further investigation [212]. Nonetheless, the encouraging results, two aspects must be considered for further development of these combinatorial approaches. In first instance, type I and II interferon signalling activates negative feedbacks restraining T cell functions (such as PD-L1 expression on myeloid and tumour cells, PD-1, and CTLA-4 expression on T cells); moreover, TLRs can be expressed by tumour cells as well and those agonists can support their proliferation potential [213, 214]. According to this, another opportunity to promote anti-tumour polarisation of myeloid cells is the employment of integrin agonists. CD11b (ITGAM), a marker shared within myeloid cells, associates with CD18 (ITGB2) to establish Mac-1 or complement receptor 3 (CR3) involved in myeloid trafficking and phagocytosis of opsonised bacteria. Recent investigations proved that CD11b has a crucial role in myeloid activation towards an inflammatory phenotype promoting tumour control in preclinical models of murine and human cancer [215]. ADH-503, a CD11b agonist, is indeed able to repolarise TAM towards an anti-tumour phenotype and to enhance dendritic cell responses which in turn support T cell-mediated tumour restriction and synergism with ICT in pancreatic cancer, a tumour in which ICT does not show any benefit when employed as single agent [67]. Given the promising preclinical results, ADH-503 recently started clinical phase 1/2 evaluation in patients with advanced solid tumour types expected to be resistant to immunotherapy, including pancreatic, prostate, breast, and MS stable colorectal cancers (clinical trial: **NCT04060342**). Finally, the p53 activation has been reported to promote MDSC differentiation to cross-presenting DCs. Indeed, the pharmacological activation of p53 drives MDSC differentiation to Ly6C^+^CD103^+^ DCs, which are essential to enhance a CD8^+^ T cell anti-tumour immune response during ICT [216]. Taken together, these recent findings pinpoint a novel therapeutic approach to induce immunosuppressive TIM differentiation to antigen-presenting cells rather than causing their elimination.

## Conclusion and future perspective

**Fig. 2:**
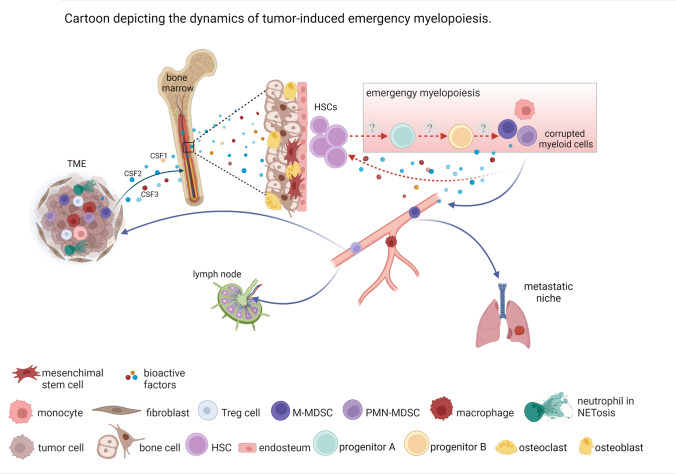
Cartoon depicting the dynamics of tumor-induced emergency myelopoiesis. Tumor microenvironment (TME)-derived soluble and bioactive factors (cytokines, growth factors, exosomes, nanoparticles, cells) condition the BM to output corrupted altered myeloid cells (emergency myelopoiesis) which promote primary cancer growth and metastatic spread (lung and lymph nodes). Hematopoietic stem cells (HSCs), in the so-called BM niche, interact with mesenchymal (MSCs) and endothelial cells that regulate HSC dormancy and differentiation into altered progenitors through cytokines and cell contact-dependent signals. Several mechanisms (depicted by dashed lines) will be the focus of future investigations. From these studies, new targets will be identified and exploited for alternative, more effective, and personalised therapeutic approaches for cancer disease

In this review, we have described our current knowledge on targets and strategies set in place to modulate the tumor-promoting function of myeloid cells. However, despite these exciting new opportunities, it is mandatory to keep in mind that all these efforts are meaningful if successfully translated to humans. In this contest, it will be important to include analysis of BM niches in which to explore potential new targets regarding MDSC generation, regulation, and trafficking. We think that targeting the BM niches presents not only an avenue to treat cancer but also inflammatory conditions, since emergency myelopoiesis is a highly regulated process in which HSC niche and external factors tilt the hematopoiesis balance towards an altered myeloid lineage. The pivotal work done by several research groups, on defining and understanding the regulatory elements sustaining HSC output, provides promising molecular targets that could potentially revert a “maladaptive” myelopoiesis into an educated one (Fig. 2). Today, single-cell omic technologies are improving the current understanding of myeloid cell biology and their contribution to tumour progression and tumour restriction. Preclinical and clinical studies highlight the importance of re-educating in spite of depleting specific myeloid cell subsets within TME in order to sustain anti-tumour immunity. From this point of view, many strategies can be enrolled to build up a new concept of cancer immunotherapy.

Recently, myeloid cell engineering by gene-editing approaches has been proposed as well to sustain antigen presentation and tumour cytotoxic activities. The manipulation of myeloid immunity has some advantages compared to T cell engineering. In first instance, myeloid cells can infiltrate TME more efficiently than lymphocytes. Secondly, engineered myeloid cells can shape TME towards a tumour restricting milieu, support priming of tumour-specific immune response, homing, and function of T lymphocytes in tumour core. For example, monocytes engineered to express a pp65 (CMV) protein fused to lysosomal-associated membrane protein (LAMP) were adoptively transferred in glioblastoma patients in order to efficiently prime a tumour-specific T cell-based immune response (clinical trial: **NCT04741984**). IL-12 and type I and II IFNs promote myeloid cell skewing towards an inflammatory phenotype: genetically engineered myeloid cells expressing IL-12 reverted the immune suppressive program in the premetastatic niche supporting tumour antigen priming and resulting in reduced metastatic and primary tumour burden in tumour-bearing mice [217]. Similar results were achieved in preclinical breast cancer setting employing IFN-γ-loaded macrophages [218]. Finally, macrophages engineered with a epidermal growth factor receptor 2 (Her2)-specific chimeric-antigen receptor showed deepen abilities to phagocyte cancer cells and secrete pro-inflammatory cytokines supporting the M1-like polarisation of bystander myeloid cells, restricting tumour burden [219].

The specific and efficient delivery of modulators to tumour-reprogrammed myeloid cells can improve the efficacy of cancer therapy. Nanoparticles (NPs) are thus excellent candidates to modulate TME-infiltrating myeloid cells [220]. NPs are carriers of any shape which size ranges between 1 and 100 nm with distinctive features for immune cell targeting such as the ability to overcome biological barriers and to be engulfed by immune cells [221]. Multiple factors impact the effectiveness of the NP-based therapy, such as (i) route of administration, (ii) particle surface charge, and (iii) drug formulation. For instance, MDSC/TAM-targeted NPs are normally infused intravenously and can accumulate passively or as consequence of myeloid cell uptake in tumours. However, systemic administration provides liver, kidney, and spleen accumulation that compromise a preferential uptake by tumour-infiltrated MDSCs/TAMs. To avoid this important limitation, several studies have developed NP-based targeting systems employing surface markers to specifically target defined immune cell subsets [222]. For instance, polyethylene glycol (PEG)-sheddable, mannose-modified NPs were developed to target M2-like TAMs via mannose-CD206 binding after pH-sensitive PEG dissociation in the acidic TME [223]. Poly-beta-amino-ester (PBAE) NPs as cargo of synthetic mRNA encoding interferon regulatory factor 5 (IRF5) was able to affect M2-like TAMs and increase the percentage of M1-like TAMs [224]. A similar TAM reprogramming is promoted also by IL-12-loaded poly-β-amino ester NPs [225]. Other NPs can be loaded with silencing molecules (i.e. shRNA, siRNA) to target crucial transcriptional factors in myeloid, reprogrammed cells such as STAT3. Indeed, the STAT3-silencing inhibited MDSC-dependent immunosuppression at the tumour site in tumour-bearing mice [226] as well as normalised the immune response by repressing plasma concentration of several pro-inflammatory cytokines in mice undergoing CRS [59]. Therefore, this approach may be tested in combination with ICT in tumour setting.

In conclusion, although we still do not completely understand the mechanisms driving innate myeloid cell polarisation towards an anti-tumour phenotype, deciphering the myeloid cell functional stages associated with worse clinical outcome and bad response to therapy will support clinicians to select the patients requiring TME reprogramming, increasing thus the therapeutic effectiveness of ICT. Strategies harnessing T cell functions and myeloid cell reprogramming will synergise in supporting immune system ability to restrict tumour progression in the next future.
